# Fungal Variation during Peanut Paste Storage

**DOI:** 10.1155/2020/8836726

**Published:** 2020-08-06

**Authors:** Zamblé Bi Irié Abel Boli, Ollo Kambire, Lessoy Thierry Zoue, Rose Koffi Nevry

**Affiliations:** ^1^Laboratory of Biotechnology and Food Microbiology, Department of Food Science and Technology, Nangui Abrogoua University, 02 BP 801, Abidjan, Côte d'Ivoire; ^2^Peleforo Gon Coulibaly University, Department of Biochemistry and Genetics, BP 1328, Korhogo, Côte d'Ivoire; ^3^Laboratory of Biotechnology, Department of Biosciences, Felix Houphouët-Boigny University, 22 Bp 582, Abidjan 22, Côte d'Ivoire

## Abstract

Peanut paste produced in multipurpose mills is very often the site of choice for fungal contaminants that pose a major risk to consumers. The objective of this study is to evaluate the level of fungal contamination of peanut paste produced according to different moulding processes during storage. Thirty samples of peanut paste were produced from 60 kg of peanut pods according to three types of moulding (domestic moulding, artisanal moulding, and hygienic moulding) and then preserved for three months. These thirty samples were subjected to microbiological analysis using the conventional mould count method. The moisture content of the various peanut pastes was determined according to the AOAC method. Fungi were identified by using taxonomic schemes based on microscopic observation and culture appearance. Mould loads ranged from 0 to 6.4.10^2^ cfu/g; 91 to 9.6.10^2^ cfu/g; and 0 to 4.6.10^2^ cfu/g, respectively, for domestic, artisanal, and hygienic mouldings during conservation. Moisture content increases during the conservation of peanut paste. It increases from 1.23 to 3.17% for domestic moulding, 1.30 to 3.20% for artisanal moulding, and 1.30 to 2.94% for hygienic moulding. Four fungal genera, namely, *Aspergillus*, *Mucor*, *Absidia*, and *Penicillium* and three species of *Aspergillus* including *A. flavus*, *A. fumigatus* and *A. niger* have been identified. The peanut paste produced from domestic and hygienic moulding is less contaminated during storage than that obtained in the artisanal way.

## 1. Introduction

Peanut is a staple food for the majority of people in sub-Saharan Africa [1, 2] and consumed in several forms, including seeds, oil, boiled, flour, cake, and paste [3, 4]. Peanut paste consumed in households is also marketed directly in markets. In Côte d'Ivoire, the control of the informal sector over the storage and marketing conditions of peanut paste production is mostly outside the control of the government. However, peanuts and their derivatives consumed in various forms are subject to contamination by pathogenic fungi throughout the production chain. The most frequent targets of these pathogenic fungi are cereals and oilseeds, particularly peanut. Peanut paste is produced in artisanal or semiartisanal production units and marketed directly in markets. Samples of peanut paste taken directly from markets in Abidjan contained various fungal genera, including *Mucor*, *Alternaria*, *Helmintosporium*, *Geotrichum*, *Fusarium*, *Cladosporium*, *Penicillium*, and *Aspergillus*, and mycotoxins, particularly aflatoxin B1 and ochratoxin A [5, 6]. Studies conducted in several sub-Saharan African countries including Mali, Benin, Nigeria, Kenya, and Côte d'Ivoire have shown contamination of peanut seeds, oil, cake, and paste by pathogenic moulds [7–9].

Several mycotoxins including aflatoxins, ochratoxin A, fumonisins, zearalenone, patulin, and many others found in food products are synthesized by moulds [10]. Among all these mycotoxins, the International Agency for Research on Cancer reported in 2002 that aflatoxin B1 causes cancer in the liver, prostate, and other human organs, while ochratoxin A is highly carcinogenic to the kidney and liver. According to [11], the level of exposure to aflatoxin B1 of Ivorian consumers of peanut paste produced in multipurpose mills varies between 2.072 ng/kg/day and 2.193 ng/kg/day and leads to a population at risk estimated between 10.1% and 15.6% compared to the tolerable daily intake of 1 ng/kg/day according to the statistical modelling of the @RISK software. The cancer risk is proven by considering the margin of exposure values for cancer which are well above the limit value of 10,000. Thus, the application of good manufacturing practice for peanut paste is necessary in order to put healthy products on the market.

It is in this context that this study was carried out in order to evaluate the action of peanut paste manufacture according to three types of moulding on fungal contamination on the one hand and the variation of the fungal load during the conservation of the peanut pastes resulting from these different mouldings on the other hand.

## 2. Materials and Methods

**Figure 1:**
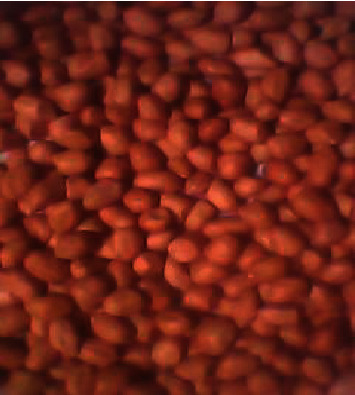
Peanut.

**Figure 2:**
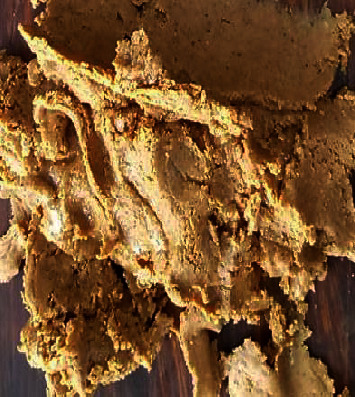
Peanut paste.

The study material consisted of peanut seeds (*Arachis hypogaea* L.) (Figure 1) from which the peanut paste samples were produced (Figure 2). These peanut seeds were collected in the sub-prefecture of Gohitafla, central-western Côte d'Ivoire.

### 2.1. Sampling of Peanut Seeds and Preparation of Peanut Paste

Sixty kilograms of dried peanut pods were collected from a groundnut production area in Gohitafla locality, Côte d'Ivoire. The peanut pods were decorticated, and the resulting seeds were roasted and bleached. The bleached peanut seeds were divided into three equal parts, and each of the three parts was moulded differently. The first part was crushed in a mortar (domestic moulding). The second part was moulded under the usual conditions of use of a multipurpose mill (artisanal moulding), and the third part was moulded in a mill and disinfected with boiling water (hygienic moulding). The peanut paste from each part has been subdivided into 12 fractions of 200 g each. Three fractions of 200 g each from each part were taken and analysed as a control. The other nine parts of each type of moulding were packed in clean, covered containers. These samples were placed in a cooler containing ice and then transported and stored in three randomly selected stores of peanut paste vendors in the markets of three communes of Abidjan (Abobo, Adjamé, and Yopougon). Samples of each type of peanut paste were taken after one month, two months, and three months of storage. A total of 36 peanut paste samples were analysed.

### 2.2. Determination of Moisture Content

The moisture content was determined according to the method of AOAC (1990) which is based on the loss of mass of the sample in the oven at 105°C until a constant mass is obtained. Thus, 5 g of peanut paste was introduced into a glass capsule of known mass (*m*_0_). The capsule containing the sample of total mass *m*_1_ has been placed in an oven (Memmert, Germany) which is set at 105 ± 2°C for a period of 24 hours. After removing from the oven, the capsule was cooled in a desiccator. After cooling, the whole content (sample + capsule) is weighed, and the mass is recorded. The operation is then repeated every 2 hours until a constant mass (*m*_2_) is obtained, and the moisture content is determined by the following formula:

(1) $$\begin{array}{c} \text{moisture content}\left(\%\right)=\frac{\left(m_{1}-m_{2}\right)}{\left(m_{1}-m_{0}\right)}\times 100. \end{array}$$

### 2.3. Mycological Analysis of Peanut Paste

The isolation of fungi was carried out according to the agar dilution method as described by [12]. Ten grams from each peanut butter sample were homogenized with 90 mL of buffer peptone water (AES Laboratory, France), and serial decimal dilutions (10^−1^ to 10^−4^) were performed. Fungal species were isolated on the semiselective Dichloran Rose Bengal Chloramphenicol (DRBC) agar (Biokar Diagnostics, France). The medium was poured into sterile Petri dish, and 0.1 mL of each sample suspension was spread-plated onto the DRBC agar in triplicate. The plates were incubated for 5 to 7 days at 25°C. Fungal isolates were subcultured on Malt Extract and Czapek Yeast medium agars (Oxoid, UK) and incubated for 5 to 7 days at 25°C for purification. Fungi were identified by using taxonomic schemes based on microscopic observation and culture appearance including colonies' colours, texture, reverse colour, hyphae arrangement, conidia shape, and nature of spores [13]. Staining with methylene blue and fuchsine was used for the microscopic observation of mould on the objective ×40. For the differentiation between *Aspergillus flavus* colonies, AFPA agar (Oxoid, UK) supplemented with chloramphenicol was used. The total fungal count for each plate was expressed as colony-forming units per gram of sample (cfu/g). For each type of moulding, the fungal contaminant density was calculated based on the storage time. Thus, the density of a fungal contaminant for a given storage time is calculated by dividing the number of that particular contaminant by the number of all contaminants present for that storage time.

### 2.4. Statistical Analysis

All the analyses were performed in triplicate, and the data were analysed using EXCELL and STATISTICA 7.1 (StatSoft). Differences between means were evaluated by Duncan's test. A significance difference was established at *α* = 0.05.

## 3. Results

### 3.1. Changes in the Moisture Content of Peanut Paste during Storage

The changes in the moisture content of the peanut paste during storage according to the method of moulding are shown in Table 1. On the first day, the moisture content was 1.23% for the domestic moulding samples and 1.3% for the artisanal and hygienic mouldings. During storage, the moisture content of the analysed samples ranged from 1.23 to 3.17% for the domestic moulding samples, from 1.3 to 3.2% and from 1.3 to 2.94% for the artisanal and hygienic mouldings, respectively.

### 3.2. Loads and Fungal Profile of Peanut Paste during Storage

#### 3.2.1. Evolution of the Fungal Load

Table 2 shows the evolution of mould loads of peanut paste samples during storage according to the method of preparation. On the first day, an absence of mould was recorded in the samples of the domestic and hygienic mouldings. On the other hand, from the beginning (first day) to the end (3 months) of the shelf life, mould loads were recorded in all of the artisanal moulding samples analysed. Mould loads ranged from 0 to 6.4.10^2^ cfu/g; 91 to 9.6.10^2^ cfu/g; and 0 to 4.6.10^2^ cfu/g, respectively, for domestic, artisanal, and hygienic mouldings during storage. The highest loads were obtained with the samples of the artisanal moulding. A significant difference (*P* < 0.05) was observed between mould loads in the peanut paste samples regardless of the moulding method.

#### 3.2.2. Fungal Profile of Peanut Paste during Storage by Moulding Methods

Based on the identification keys, four fungal genera, namely, *Muco*r, *Absidia*, *Penicillium*, *Aspergillus*, and three species of *Aspergillus* were tentatively identified as *A. flavus*, *A. fumigatus*, and *A. niger*.  Table 3 shows the evolution of these fungal contaminants identified in peanut paste samples during storage according to the type of moulding. On the first day, *Mucor* sp. was the only fungal contaminant isolated in the artisanal moulding samples. *A. flavus* and *A. niger* were the only species of *Aspergillus* that appeared in the samples analysed regardless of the type of moulding from the first month of storage. *Penicillium* sp. (domestic moulding), *Absidia* sp. and *Penicillium* sp. (artisanal moulding), and *Mucor* sp (hygienic moulding) which were absent in the first month of storage appeared from the second month onwards. All the same fungal contaminants from the second month appeared in the third month of storage. *A. niger* was the only species of *Aspergillus* that appeared in all samples tested regardless of the moulding type. The number of *Aspergillus* species increased from two (*A. flavus* and *A. niger*) at the first month to three (*A. flavus*, *A. niger*, and *A. fumigatus*) at the second month in the artisanal moulding samples. In the domestic moulding samples, the number of *Aspergillus* species increased from two (*A. flavus* and *A. niger*) in the second month of storage to one (*A. niger*) in the third month. *A. niger* was the only species isolated in the hygienic moulding samples.

#### 3.2.3. Density of Fungal Contaminant Isolation by Moulding Type during Storage

Apart from the artisanal moulding paste, no fungal genus was identified on the first day in the other pastes according to the type of moulding. In the peanut paste of the domestic moulding, *Aspergillus flavus* was most identified after one month of storage; in the second month, the density was identical for *A. flavus*, *A. niger*, and *Penicillium* sp. (33%). After three months of storage, an absence of *A. flavus* was observed (Table 4).

Concerning the peanut paste from the artisanal moulding, *Mucor* sp. and the species of *A. flavus* and *A. niger* were the most identified (33%) in the second month of storage; *Mucor* sp. and *A. niger* were the most abundant (25%) in the second month of storage. In the third month of storage, the most abundant fungal strains were *Mucor* sp., *Penicillium* sp., *A. niger*, and *A. flavus* with identical densities of 20%.

In the peanut paste of the hygienic moulding, two fungal strains (*A. niger*, *Mucor* sp) were identified with a dominance of *A. niger* after 1 month (100%) and 3 months (67%) of storage. After two months of storage, the density (50%) is identical for both fungal strains.

## 4. Discussion

The peanut paste obtained from the different types of moulding was kept in closed plastic containers. The structure of these containers does not allow the passage of water from the atmosphere into the product (peanut paste). Thus, the moisture content of the peanut paste samples varied somewhat during storage. The results obtained during the first two months of storage are consistent with those obtained (1.8%) by [14] in the peanut paste used in Pakistan. Throughout the storage period, the moisture content of the samples remained below the critical value of 8%, above which there may be abundant mould growth [15]. The low moisture content observed in the analysed samples on the first day could be related to good drying and/or roasting of the peanut seeds which are used to make peanut paste as reported by [16, 17]. The technique used for this study measures evaporation at high temperature. Thus, what was detected as moisture after the first, second, and third month of storage could be due to volatile compounds from the enzymatic degradation of lipids or other compounds in the peanut paste. However, peanut pastes should be stored in a well-ventilated environment to avoid absorption of moisture from the air during storage. According to [18, 19], good air circulation in storage can prevent absorption of moisture from the immediate environment.

Peanut paste samples obtained from domestic and hygienic moulding are less loaded than those from artisanal moulding. Various food products are moulded in the artisanal mills. These different products after moulding leave residues in the mill. These nutrient-rich residues make the mill a favourable environment for the proliferation of microorganisms. Thus, after the formation of this microflora in the mill, all food passing through the mill is likely to be contaminated. With the hygienic moulding technique, the disinfection of the mill with boiling water before moulding allows the elimination of several microorganisms resulting in the reduction of mould loads in the peanut pastes from hygienic moulding compared to those from artisanal moulding. Peanut pastes from domestic moulding were likely contaminated with mortar and airborne mould spores. The results observed during the three months of storage regardless of the preparation method are consistent with those obtained by [7] in peanut cakes in Benin. These results are below the maximum level (10^4^ cfu/g) recommended by the International Commission on Food Microbiology for peanut butters or pastes. These results could be explained by the good conditions under which the peanut paste is made, including seed sorting, domestic and hygienic moulding, clean and covered containers, and transportation of the peanut paste samples under the recommended conditions (cooler containing ice). Indeed, according to [20], practices such as sorting help reduce fungal contamination of products. In addition, Mutegi [21] reported that poor transportation conditions and marketing of peanut products or cakes can contribute to mould growth.

Fungal contaminants that have been identified in peanut paste samples analysed during storage are *Mucor* sp., *Absidia* sp., *Penicillium* sp., *A. flavus*, *A. niger*, and *A. fumigatus*. The results of this study are similar to the work of [8] who isolated most of these fungal strains, including *A. flavus*, *A. parasiticus*, *A. tamarii*, *A. alliaceus*, *A. fumigatus*, *A. niger, Fusarium*, *Mucor* sp., *Penicillium* sp., and *Rhizopus* sp., from peanut paste samples in the provinces of Kenya. The contaminants identified in this study belong to storage fungi that grow on food during storage [22, 23]. *Aspergillus* was the only fungal genus that were progressively isolated from all the samples analysed, regardless of the method of preparation. This is consistent with the work of several authors [24–26]. They reported the progressive increase of fungi of the genus *Aspergillus* during storage periods of peanuts and peanut products. The proliferation of *Aspergillus* in peanut paste during storage may be related to the use of carbohydrates, protein, fibre, and fat as growth-promoting nutrients [27, 28].

*A. flavus* and *A. niger* have been the main species isolated from peanut pastes during storage regardless of the method of preparation. This result is in accordance with the work of several authors who have also reported that *A. flavus* and *A. niger* are the major storage fungi [29, 30]. The predominance of *A. flavus* and *A. niger* in analysed samples may pose a public health concern given their ability to produce mycotoxins [31, 32]. According to [33], *A. flavus* can produce aflatoxins. On the other hand, the authors of [34, 35] reported the production of ochratoxin A from *A. niger* in food.

The species *Aspergillus flavus* is absent after three months of storage in peanut paste from domestic moulding. This inhibition could be related to the action of bacteria. Indeed, Xianfeng [36] reported the antifungal action of several bacteria (*Bacillus*, *Lactobacillus*, *Streptomyces* etc.) on *A. flavus*. According to these same authors, *A. niger* has an inhibitory effect on the growth of *A. flavus*.

## 5. Conclusion

Peanut paste samples obtained from domestic and hygienic moulding are less susceptible to fungal growth during storage than those obtained from artisanal moulding. This study showed that samples of peanut paste produced from different moulding processes and subjected to storage are minimally contaminated with moulds such as *Mucor* sp., *Absidia* sp., *Penicillium* sp., and *Aspergillus*. The dominant contaminants belong to the genus *Aspergillus*, including species of *A. flavus* and *A. niger*. In addition, the presence of pathogenic fungi including *A. flavus*, *A. niger*, and *Penicillium* sp. is a health risk for consumers of peanut paste especially since these moulds are the potential producers of aflatoxin B1 and ochratoxin A in food. To ensure better quality of the peanut paste, owners of artisanal mills must necessarily do complete cleaning and disinfection of their mill after each moulding of the food product. Vendors must keep peanut paste in cold storage.

## Figures and Tables

**Table 1:** Changes in the moisture content of peanut paste during storage according to the moulding method.

Moulding method	Moisture content (%)			
	First day	1 month	2 months	3 months
Domestic moulding	1.23 ± 0.25^a^	1.47 ± 0.06^a^	1.93 ± 0.25^a^	3.17 ± 1.71^a^
Artisan moulding	1.3 ± 0.1^a^	1.6 ± 0.06^a^	2.16 ± 0.6^ba^	3.2 ± 1.71^ba^
Hygienic moulding	1.3 ± 0.1^a^	1.5 ± 0.1^a^	1.7 ± 0.3^a^	2.94 ± 0.1^ba^

The values assigned to the same letter are not significantly different at the 5% threshold according to Duncan's test.

**Table 2:** Evolution of the mould load of peanut paste samples during storage according to the method of moulding.

Moulding method	Peanut paste mould load (cfu/g)			
	First day	1 month	2 months	3 months
Domestic moulding	—	4.6 ± 1.3.10^1ba^	3.2 ± 0.4.10^2cb^	6.4 ± 0.3.10^2db^
Artisan moulding	91^ab^	1.8 ± 1.7.10^2bc^	5.9 ± 0.2.10^2c^	9.6 ± 0.1.10^2dc^
Hygienic moulding	—	9.1 ± 1.7.10^1b^	2.7 ± 0.5.10^2ca^	4.6 ± 0.1.10^2da^

The values assigned to the same letter are not significantly different at the 5% threshold according to Duncan's test.

**Table 3:** Evolution of fungal contaminants identified in peanut paste samples during storage according to the type of moulding.

Shelf life (months)	Fungal genera		
	Domestic moulding	Artisanal moulding	Hygienic moulding
First day	ND	*Mucor* sp	ND
1 month	*A. flavus*	*A. flavus, A. niger, Mucor* sp.	*A. niger*
2 months	*A. flavus, A. niger,* and *Penicillium* sp.	*A. flavus, A. niger, A. fumigatus*, *Mucor* sp.*, Absidia* sp., and *Penicillium* sp.	*A. niger, Mucor* sp
3 months	*A. niger* and *Penicillium* sp.	*A. flavus, A. niger, A. fumigatus*, *Mucor* sp*, Absidia* sp, and *Penicillium* sp.	*A. niger, Mucor* sp

ND = Not detected; A. = *Aspergillus*.

**Table 4:** Density of fungal contaminants.

Moulding method	Fungal contaminants	Density (percentage)			
		First day	1 month	2 months	3 months
Domestic moulding	*A. flavus*	0	100	33	0
	*A. niger*	0	0	33	50
	*Penicillium* sp.	0	0	33	50
Artisanal moulding	*Mucor* sp.	100	33	25	20
	*Absidia* sp.	0	0	12	10
	*Penicillium* sp.	0	0	12	20
	*A. flavus*	0	33	12	20
	*A. niger*	0	33	25	20
	*A. fumigatus*	0	0	12	10
Hygienic moulding	*Mucor* sp.	0	0	50	33
	*A. niger*	0	100	50	67

## Data Availability

Data used to support the findings of this study are included within the article.
